# Supplementary material for: Prevalence, determinants, and antimicrobial susceptibility patterns of Campylobacter infection among under-five children with diarrhea at Governmental Hospitals in Hawassa city, Sidama, Ethiopia. A cross-sectional study
Source: PLoS One. 2022 May 11;17(5):e0266976. doi: 10.1371/journal.pone.0266976 (PMC9094509; doi:10.1371/journal.pone.0266976)
Supplement: Attachment — Submitted filename: Point by point Response P.docx [file pone.0266976.s001.docx]

CC: abdelazeem.algammal@vet.suez.edu.eg, abdelazeem.algammal@gmail.com

PONE-D-22-01816

Prevalence, antimicrobial susceptibility patterns and associated factors of Campylobacter infection among under-five children with diarrhea at Governmental Hospitals in Hawassa city, Sidama, Ethiopia. A cross-sectional study.

PLOS ONE

Dear Dr. Fenta,

Thank you for submitting your manuscript to PLOS ONE. After careful consideration, we feel that it has merit but does not fully meet PLOS ONE’s publication criteria as it currently stands. Therefore, we invite you to submit a revised version of the manuscript that addresses the points raised during the review process.

ACADEMIC EDITOR: Please revise the manuscript according to the reviewer comments, a major revision is required. The manuscript should be revised for English editing and grammar mistakes.

Please submit your revised manuscript by Mar 11 2022 11:59PM. If you will need more time than this to complete your revisions, please reply to this message or contact the journal office at [plosone@plos.org](mailto:plosone@plos.org). When you're ready to submit your revision, log on to https://www.editorialmanager.com/pone/ and select the 'Submissions Needing Revision' folder to locate your manuscript file.

Please include the following items when submitting your revised manuscript:

- A rebuttal letter that responds to each point raised by the academic editor and reviewer(s). You should upload this letter as a separate file labeled 'Response to Reviewers'.
- A marked-up copy of your manuscript that highlights changes made to the original version. You should upload this as a separate file labeled 'Revised Manuscript with Track Changes'.
- An unmarked version of your revised paper without tracked changes. You should upload this as a separate file labeled 'Manuscript'.

If you would like to make changes to your financial disclosure, please include your updated statement in your cover letter. Guidelines for resubmitting your figure files are available below the reviewer comments at the end of this letter.

If applicable, we recommend that you deposit your laboratory protocols in protocols.io to enhance the reproducibility of your results. Protocols.io assigns your protocol its own identifier (DOI) so that it can be cited independently in the future. For instructions see: <https://journals.plos.org/plosone/s/submission-guidelines#loc-laboratory-protocols>. Additionally, PLOS ONE offers an option for publishing peer-reviewed Lab Protocol articles, which describe protocols hosted on protocols.io. Read more information on sharing protocols at <https://plos.org/protocols?utm_medium=editorial-email&utm_source=authorletters&utm_campaign=protocols>.

We look forward to receiving your revised manuscript.

Kind regards,

Abdelazeem Mohamed Algammal, Prof, Ph.D
Academic Editor
PLOS ONE

**Cover letter**

**DEAR: JOURNAL PLOSE ONE EDITORIAL OFFICE.**

We are very grateful to editors who have critically revised the manuscript for the first time and provided and raised a number of points that we believe would improve the manuscript and may allow us with points to improve its quality. Therefore, we have incorporated each and every comment of the Editors and reviewers in the revised manuscript with highlighting with yellow color. Moreover, point-by-point responses are given below to the raised queries for editors

We hope that each and every point is now addressed to the satisfaction of the Editors and reviewers

We look forward to hearing from you soon.

Kind Regards

**Title:** Prevalence, antimicrobial susceptibility patterns and associated factors of Campylobacter infection among under-five children with diarrhea at Governmental Hospitals in Hawassa city, Sidama, Ethiopia. A cross-sectional study.

PONE-D-22-01816

Journal Requirements:

When submitting your revision, we need you to address these additional requirements.

1. Please ensure that your manuscript meets PLOS ONE's style requirements, including those for file naming. The PLOS ONE style templates can be found at
   https://journals.plos.org/plosone/s/file?id=wjVg/PLOSOne_formatting_sample_main_body.pdf and 
   <https://journals.plos.org/plosone/s/file?id=ba62/PLOSOne_formatting_sample_title_authors_affiliations.pdf>

**Response:** The comment is well taken and agrees with the Editor’s comment, more ever we tried to read and follow the PLOSE ONE style template as much as possible.

2. Thank you for stating the following financial disclosure:

"The authors have not declared a specific grant for this research from any funding agency in the public, commercial or not-for-profit sectors.

The funders had no role in study design, data collection, and analysis, decision to publish, or preparation of the manuscript"

At this time, please address the following queries:

a) Please clarify the sources of funding (financial or material support) for your study. List the grants or organizations that supported your study, including funding received from your institution.

**Response:** The comment is also well taken again and we are sure that no kind of fund would be secured from any funding organization and other partners, except material support and perdiaum for data collectors was supported by Hawassa University College of Medicine and health science. This was explained in the supporting information uploaded during submission. Therefore: “The funders had no role in study design, data collection, and analysis, decision to publish, or preparation of the manuscript.”
b) State what role the funders took in the study. If the funders had no role in your study, please state: “The funders had no role in study design, data collection, and analysis, decision to publish, or preparation of the manuscript.”
c) If any authors received a salary from any of your funders, please state which authors and which funders.

**Response:** The comment is well taken again and we are sure that no kind of fund would be secured from any funding organization and other partners, except support by Hawassa University College of Medicine and health science for material support and perdiaum for data collectors. This was explained in the cover letter uploaded during submission. Therefore; “Any of the authors were not received a salary from any of the funders”
d) If you did not receive any funding for this study, please state: “The authors received no specific funding for this work.”

Please include your amended statements within your cover letter; we will change the online submission form on your behalf.

**Response:** The comment is well taken again and we are sure that no kind of fund would be secured from any funding organization and other partners, except support by Hawassa University College of Medicine and health science for material support and perdiaum for data collectors. This was explained in the cover letter uploaded during submission.

[Note: HTML markup is below. Please do not edit.]

**Cover letter**

**DEAR: JOURNAL PLOSE ONE REVIEWERS.**

We are very grateful to editors who have critically revised the manuscript for the first time and provided and raised a number of points that we believe would improve the manuscript and may allow us with points to improve its quality. Therefore, we have incorporated each and every comment of the **Reviewers** in the revised manuscript with highlighting with yellow color. Moreover, point-by-point responses are given below to the raised queries for **Reviewers.**

We hope that each and every point is now addressed to the satisfaction of the Reviewers

We look forward to hearing from you soon.

Kind Regards

**Title:** Prevalence, antimicrobial susceptibility patterns and associated factors of Campylobacter infection among under-five children with diarrhea at Governmental Hospitals in Hawassa city, Sidama, Ethiopia. A cross-sectional study.

PONE-D-22-01816

Reviewers' comments:

Reviewer's Responses to Questions

**Comments to the Author**

1. Is the manuscript technically sound, and do the data support the conclusions?

The manuscript must describe a technically sound piece of scientific research with data that supports the conclusions. Experiments must have been conducted rigorously, with appropriate controls, replication, and sample sizes. The conclusions must be drawn appropriately based on the data presented.

Reviewer #1: Partly

**Response:** We agree with the reviewer’s comment and we tried to correct based on the comment

Reviewer #2: Yes

**Response:** The comment is well taken and we thank you for your encouraging comment.

2. Has the statistical analysis been performed appropriately and rigorously?

Reviewer #1: Yes

**Response:** The comment is well taken and we thank you for your encouraging comment.

Reviewer #2: Yes

**Response:** The comment is well taken and we thank you for your encouraging comment.

3. Have the authors made all data underlying the findings in their manuscript fully available?

**Response:** The comment is well taken and all data underlying the findings are in our manuscript. Finally we included it in data availability section of the availability.

The [PLOS Data policy](http://www.plosone.org/static/policies.action#sharing) requires authors to make all data underlying the findings described in their manuscript fully available without restriction, with rare exception (please refer to the Data Availability Statement in the manuscript PDF file). The data should be provided as part of the manuscript or its supporting information, or deposited to a public repository. For example, in addition to summary statistics, the data points behind means, medians and variance measures should be available. If there are restrictions on publicly sharing data—e.g. participant privacy or use of data from a third party—those must be specified.

Reviewer #1: No

**Response:** The comment is well taken and all data underlying the findings are in our manuscript. Finally, we included it in the data availability in the additional information sheet

Reviewer #2: Yes

**Response:** The comment is well taken and we thank you for your encouraging comment.

4. Is the manuscript presented in an intelligible fashion and written in standard English?

PLOS ONE does not copyedit accepted manuscripts, so the language in submitted articles must be clear, correct, and unambiguous. Any typographical or grammatical errors should be corrected at revision, so please note any specific errors here.

Reviewer #1: No

**Response:** We agree with the reviewer comment and concern and tried to revise the language and punctuation throughout the manuscript according to the given comment

Reviewer #2: No

**Response:** We agree with the reviewer comment and concern and tried to revise the language and punctuation throughout the manuscript according to the given comment

5. Review Comments to the Author
Please use the space provided to explain your answers to the questions above. You may also include additional comments for the author, including concerns about dual publication, research ethics, or publication ethics. (Please upload your review as an attachment if it exceeds 20,000 characters)

Reviewer #1: Comments to authors:
-The current study is interesting; however, the authors should address the following comments to improve the quality of the manuscript
Title:
I think the work would benefit from the title that contains the main conclusion of the study (should be derived from the conclusion). Please modify the title.

**Response:** We agree with the reviewer’s comment and tried to modify it according to the comment given and also tried to link with a conclusion.
Abstract:
- The abstract must illustrate the used methods and the most prevalent results (give more hints about methods and results).

**Response:** We agree with the reviewer’s comment and concern and tried to revise the abstract section to mention the methods and the most prevalent results (line #23-55)

Besides, rephrase the aim of the work and the main conclusion of your findings.

**Response:** We agree with the reviewer’s comment and concern and tried to rephrase the abstract section to link with the conclusion part.
Introduction: (it needs to be more informative)

**Response:** We agree with the reviewer’s comment and tried to modify it according to the comment given.
-Give a hint about the virulence factors, different infections caused by Campylobacter, and the mechanism of disease occurrence.

**Response:** We agree with the reviewer’s comment and tried to modify it according to the comment given from Line # 92-110 in the manuscript
- The authors should illustrate the public health importance concerning the emergence of multidrug-resistant (MDR) bacterial pathogens that reflect the necessity of new potent and safe antimicrobial agents. Several studies proved the widespread MDR- bacterial pathogens;
Authors could add the following paragraph:

Multidrug resistance has been increased all over the world that is considered a public health threat. Several recent investigations reported the emergence of multidrug-resistant bacterial pathogens from different origins including humans, birds, cattle, and fish that increase the need for routine application of the antimicrobial susceptibility testing to detect the antibiotic of choice as well as the screening of the emerging MDR strains. You should cite the following valuable studies:

1.PMID: 33177849
2.PMID: 33188216
3.PMID: 30150182
4.PMID: 33947875
5.PMID: 32994450
6. PMID: 32497922
7.PMID: 33061472
8.PMID: 34445951
9.https://doi.org/10.1016/j.aquaculture.2021.737643

10.https://doi.org/10.1016/j.foodcont.2021.108066

**Response:** We agree with the reviewer’s comment and tried to address each issue as given in the comment in the main manuscript from line (#107-126), however, most of the references are not specifically related to our specific topic
-Rephrase the aim of the work to be clear and better sound.

**Response:** We agree with the reviewer’s comment and tried to rephrase the manuscript on the aim of the study based on the comment and to the best of our manuscript quality Line (#135-156).
Material and methods:
-Add this subtitle: Bacterial Isolation and identification:

**Response:** We agree with the reviewer’s comment and tried to subtitle with the given comment in the methods section line (#221)
•Discuss in detail the methods of isolation and identification of Campylobacter. Besides, specific references should be added.

**Response:** We agree with the reviewer’s comment and tried to discuss the methods part in detail on the isolation and identification of Campylobacter with specific references in the methods section from lines # 221-246 based on the comment and to the best of our manuscript quality.
•Add the company, city, and country of the used bacterial media and reagents that were used in the biochemical identification of isolates. Also, enumerate all used biochemical reactions.

**Response:** We agree with the reviewer’s comment and tried to address the country of the media and biochemical tests used in the current study (line # 139-142).

-Antimicrobial susceptibility testing:
•Add the names of the antimicrobial classes of the tested antibiotics.

**Response:** We agree with the reviewer’s comment and tried to address the country of the media and biochemical tests used in the current study (line # 139-142).

•The authors are advised to classify the tested isolates to MDR , XDR, and PDR as described by Magiorakos et al.
Magiorakos AP, Srinivasan A, Carey RB, Carmeli Y, Falagas ME, Giske CG, et al. Multidrug-resistant, extensively drug-resistant and pandrug-resistant bacteria: An international expert proposal for interim standard definitions for acquired resistance. Clin Microbiol Infect. 2012; 18:268–81. doi:10.1111/j.1469-0691.2011.03570.x.

- PCR-based detection of the antimicrobial resistance genes should be performed. Afterward, the correlation between phenotypic and genotypic multidrug resistance should be performed.

**Response:** We agree with the reviewer’s comment and for the future we have plan to conduct a large scale survey using PCR-based detection the correlation between phenotypic and genotypic multidrug resistance will be performed in the future. In this study, we tried to mention it as one of the limitations of the study in the discussion section.

-Statistical analyses:-Add more details about the software used in the statistical analyses.

**Response:** We agree with the reviewer’s comment and added a few details about the SPSS software that we use for analysis if we tried to understand the reviewer’s raised issue in the data processing and analysis section of the manuscript line (#293-295).

-Results:
-Add this subtitle: Phenotypic characteristics of the recovered isolates:
•Illustrate in detail the phenotypic characteristics of the recovered Campylobacter isolates.

**Response:** We agree with the reviewer’s comment and added details of the phenotypic identification techniques that we used in this study. However; the details of the methods of isolation is also indicated in the methods part of the manuscript line (#335-351).

-Antimicrobial susceptibility testing:
•-Illustrate in a new table the occurrence of MDR (Multidrug Resistance) among the recovered isolates as the following (illustrate the names of the antimicrobial classes and different antibiotics): No. of strains%. Type of resistance R, MDR, and XDR Phenotypic multidrug resistance (Antimicrobial classes and different antibiotics).The antibiotic-resistance genes

**Response:** We agree with the reviewer’s comment and tried to address the classes of antibiotics used in this study in **Table 5.** And R, MDR, and XDR Phenotypic multidrug resistance (Antimicrobial classes and different antibiotics) in Table 6

The antibiotic-resistance genes
-The correlation (Correlation coefficient) between phenotypic and genotypic multidrug resistance should be performed.-You should support your results with illustrating figures.
**Response:** We agree with the reviewer’s comment and for the future we have plan to conduct a large-scale survey using PCR-based detection the correlation between phenotypic and genotypic multidrug resistance will be performed in the future. In this study, we tried to mention it as one of the limitations of the study in the discussion section.

-Discussion:
- The authors are advised to illustrate the real impact of their findings.
-Illustrate the different mechanisms of antimicrobial resistance in Campylobacter.

**Response:** We agree with the reviewer’s comment and we tried to rephrase and show the real impact of the findings in some parts of the discussion.

-Conclusion
- Should be rephrased to be sounded. A real conclusion should focus on the question or claim you articulated in your study, which resolution has been the main objective of your paper?

**Response:** We agree with the reviewer’s comment and we tried to rephrase the conclusion based on the comment given to link with the finding.

Reviewer #2: Comments to authors:
- The current study has a significant impact, but it needs a major revision:
- The manuscript should be revised for grammar mistakes.

**Response:** We agree with the reviewer’s comment and we tried to rephrase copy edit, topographical, and grammar mistakes throughout the manuscript based on the comment and to the best of our manuscript quality.

- Please write the scientific names of bacterial pathogens and genes in the correct form all over the manuscript and in the References section (should be italic).

**Response:** We agree with the reviewer’s comment and concern and tried to revise the abstract section to link with the conclusion part.
-The title is broad, please modify the title.

**Response:** We agree with the reviewer’s comment and concern and tried to revise the abstract section to link with the conclusion part.
- Add more details about the used methods and most prevalent results in the abstract.

**Response:** We agree with the reviewer’s comment and concern and tried to revise the abstract section to link with the conclusion part.
-In the introduction: discuss the public health importance of Campylobacter spp. and their virulence determinants.

**Response:** We agree with the reviewer’s comment and concern and tried to revise the abstract section to link with the conclusion part.
-Improve the aim of work.
**Response:** We agree with the reviewer’s comment and tried to rephrase the manuscript on the aim of the study based on the comment and to the best of our manuscript quality Line (#135-156).

Methods:
-Explain the methods of isolation and identification in detail??

-Specific references should be added to all the used methods and techniques.

**Response:** We agree with the reviewer’s comment and tried to discuss the methods part in detail on the isolation and identification of Campylobacter with specific references in the methods section from line # 246-276 based on the comment and to the best of our manuscript quality
- Antimicrobial susceptibility testing:

Add the manufacturing company, city, and country for the used reagents and antimicrobial discs.

**Response:** We agree with the reviewer’s comment and tried to address the country of the media and biochemical tests used in the current study (line # 139-142).

-PCR based detection of virulence genes and antimicrobial resistance genes in the most prevalent retrieved bacterial species should be carried out if applicable (or addresses this point in the study limitations)

**Response:** We agree with the reviewer’s comment and for the future, we have a plan to conduct a large-scale survey using PCR-based detection the correlation between phenotypic and genotypic multidrug resistance will be performed in the future. In this study, we tried to mention it as one of the limitations of the study.

-Results:
- Discuss in detail the phenotypic characters of the recovered isolates.
**Response:** We agree with the reviewer’s comment and added details of the phenotypic identification techniques that we used in this study. However; the details of the methods of isolation is also indicated in the methods part of the manuscript line (#335-351).
-PCR based detection of virulence genes and antimicrobial resistance genes in the retrieved isolates should be carried out if applicable (or addresses this point in the study limitations)
-The correlation between the phenotypic and genotypic MDR should be performed.
**Response:** We agree with the reviewer’s comment and for the future, we have a plan to conduct a large scale survey using PCR-based detection the correlation between phenotypic and genotypic multidrug resistance will be performed in the future. In this study, we tried to mention it as one of the limitations of the study in the discussion section.

-Discussion:
- Please improve.

**Response:** We agree with the reviewer’s comment and we tried to rephrase and show the real impact of the findings in some parts of the discussion.

-Please improve the main conclusion of the manuscript.

**Response:** We agree with the reviewer’s comment and we tried to rephrase the conclusion based on the comment given to link with the finding.

6. PLOS authors have the option to publish the peer review history of their article ([what does this mean?](https://journals.plos.org/plosone/s/editorial-and-peer-review-process#loc-peer-review-history)). If published, this will include your full peer review and any attached files.

If you choose “no”, your identity will remain anonymous but your review may still be made public.

**Do you want your identity to be public for this peer review?** For information about this choice, including consent withdrawal, please see our [Privacy Policy](https://www.plos.org/privacy-policy).

Reviewer #1: No

Reviewer #2: No

[NOTE: If reviewer comments were submitted as an attachment file, they will be attached to this email and accessible via the submission site. Please log into your account, locate the manuscript record, and check for the action link "View Attachments". If this link does not appear, there are no attachment files.]

While revising your submission, please upload your figure files to the Preflight Analysis and Conversion Engine (PACE) digital diagnostic tool, <https://pacev2.apexcovantage.com/>. PACE helps ensure that figures meet PLOS requirements. To use PACE, you must first register as a user. Registration is free. Then, login and navigate to the UPLOAD tab, where you will find detailed instructions on how to use the tool. If you encounter any issues or have any questions when using PACE, please email PLOS at [figures@plos.org](mailto:figures@plos.org). Please note that Supporting Information files do not need this step.

*In compliance with data protection regulations, you may request that we remove your personal registration details at any time.* [*(Remove my information/details)*](https://www.editorialmanager.com/pone/login.asp?a=r)*. Please contact the publication office if you have any questions.*
